# Niche Expansion Has Increased the Risk of *Leptocybe invasa* Fisher *Et* LaSalle Invasions at the Global Scale

**DOI:** 10.3390/insects15120985

**Published:** 2024-12-12

**Authors:** Xianheng Ouyang, Jiangling Pan, Hui Rao, Qiaoyun Sun

**Affiliations:** 1 College of Forestry, Northwest A&F University, Yangling 712100, China; oyxh@nwafu.edu.cn; 2 Zhejiang Provincial Forestry Fund Management Center, Hangzhou 310000, China; 13868026648@163.com; 3 Shaanxi Province Ankang City Langao County Forestry Bureau, Ankang 725400, China; sosrh@163.com; 4 School of Architecture and Urban Planning, Shenzhen University, Shenzhen 518060, China

**Keywords:** invasive alien species, *Leptocybe invasa*, optimized MaxEnt model, environmental variables, niche dynamics, potential geographical distribution

## Abstract

Invasive alien species often undergo shifts in their ecological niches when they establish themselves in environments that differ from their native habitats. *Leptocybe invasa* Fisher *et* LaSalle (Hymenoptera: Eulophidae), specifically, has caused huge economic losses to *Eucalyptus* trees in Australia. The global spread of *eucalyptus* cultivation has allowed *L. invasa* to threaten plantations beyond its native habitat. It is, therefore, urgent to implement effective control measures to mitigate the impact of this pest. The optimized MaxEnt model was used to predict the potential global distribution of *L. invasa* based on occurrence data and environmental variables. The centroid shift, overlap, unfilling, and expansion (COUE) framework was employed to evaluate niche dynamics during the global invasion process by comparing the ecological niches of *L. invasa* in both native regions and regions affected by invasions (hereafter referred to as “invaded”). The results indicated that the distribution of *L. invasa* is primarily influenced by temperature, precipitation, and the human influence index variables. Its ecological niche was shown to have considerably expanded from native to invaded regions. Under future climate scenarios, the potential geographical distribution of *L. invasa* is projected to be concentrated primarily in East Asia, Southeast Asia, Western Europe, and Southern Oceania. In the future, the potentially suitable areas for the establishment of *L. invasa* are expected to further expand. This study provides a unified framework for exploring the niche dynamics of invasive alien species globally. Emphasizing early warning and control in uninvaded areas is crucial for minimizing *L. invasa* ecological and economic threats.

## Simple Summary

Invasive alien species, such as *Leptocybe invasa* Fisher *et* LaSalle (Hymenoptera: Eulophidae), often adapt to new environments, causing significant damage to native ecosystems. *L. invasa* has led to major economic losses in *Eucalyptus* plantations in Australia and is spreading globally. To predict its potential distribution, the MaxEnt model was applied using occurrence data and environmental factors. This study found that the pest’s distribution is influenced by temperature, precipitation, and human activity. Its ecological niche has expanded from native to invaded regions. Projections indicate that *L. invasa* will spread further in East Asia, Southeast Asia, Western Europe, and Southern Oceania under future climate conditions. This research emphasizes the need for early detection and control measures to mitigate ecological and economic impacts of this invasive species.

## 1. Introduction

A growing body of evidence suggests that in the Anthropocene, human activities (or even the mere presence of humans) directly and indirectly influence species distributions. Notable examples include population growth, species invasions, urban expansion, and land-use changes [1]. Human activities have accelerated biodiversity loss, driving shifts in species ranges, including contractions, expansions, and relocations. When predicting species distributions, overlooking the human influence index (HII) can result in biased model outcomes and undermine the effectiveness of conservation efforts [1]. Globalization has led to increased trade and tourism between countries, facilitating the frequent movement of people and goods. This high-frequency exchange has inadvertently promoted biological invasions [2]. These events are a major driver of biodiversity loss, causing serious negative impacts on native species, habitats, and ecosystems [3], and they are considered one of the top ten global environmental issues [4]. Understanding the processes of biological invasion, considering both invasive and native species, is crucial for identifying and predicting potential invasion areas as well as for developing effective prevention and control strategies [5]. In the context of biological invasions, invasive species often compete with native ones that occupy similar ecological niches [6]. This competition can consistently reduce the abundance of native species across their range, potentially leading to competitive exclusion and even extinction [7]. The degree of competition between species can be measured by niche overlap, which quantifies the impact of invasive species on native ones with similar ecological niches [8]. This information is critical for informing policy decisions and management strategies aimed at mitigating the effects of invasive species [9].

Species distribution models (SDMs) assume that a species ecological niche remains relatively stable across space and time. However, while many studies have shown a degree of conservatism in the climatic niches of invasive species, evidence suggests that some invaders can overcome the ecological constraints of their native ranges when colonizing new habitats. This adaptability, driven by increased ecological plasticity and exposure to novel natural selection pressures, results in adaptive niche shifts in the invaded territories, as exemplified by the expansion of the niche of the floating plant *Myriophyllum aquaticum* (Vell.) Verdc. (Haloragaceae) [10]. In China, the suitable habitat areas for invasive plants such as *Ageratina adenophora* (Asteraceae), *Alternanthera philoxeroides* (Mart.) Grisb. (Amaranthaceae), *Ambrosia artemisiifolia* (Asteraceae), and *Mikania micrantha* Kunth (Asteraceae) are projected to significantly expand under future climate scenarios, with varying levels of increase and a general northward shift in their habitat centers [11]. Integrating species occurrence records with the environmental data of their surrounding habitats is considered the most effective and feasible approach for predicting species’ geographic ecological niches. Environmental changes will result in spatial range shifts when projecting their ecological niche into geographic space. SDMs utilize statistical and machine learning algorithms to explore the relationship between species occurrence locations and their environmental factors (i.e., covariates, variables, and parameters). This enables the prediction of species’ occurrence probabilities (or habitat suitability) across geographic space and/or time. The relationships between species and the environment derived from SDMs also reveal the multidimensional environmental gradients occupied by a species, known as the hypervolume niche. This capability has made SDMs widely applicable in fields ranging from disease ecology to conservation biology [12,13]. This integrated approach enhances the understanding of niche shifts and provides more realistic projections of dispersal dynamics and invasion potential.

*Eucalyptus* is one of the most commercially significant tree species, with plantations spanning tropical, subtropical, and temperate regions worldwide [14]. However, the growth of various *eucalyptus* species is threatened by *Leptocybe invasa* Fisher *et* LaSalle (Hymenoptera: Eulophidae), an invasive pest that damages trees by feeding on their tender leaves and shoots [15]. *L. invasa* lays its eggs in eucalyptus tissues, where the larvae induce gall formation for protection [15]. After developing within the galls, the larvae emerge as adults, continuing the life cycle, which allows the pest to thrive in favorable environments [16]. Particularly in warm, humid habitats with abundant eucalyptus hosts, *L. invasa* has spread rapidly, influenced by climate, host availability, and the absence of natural predators. Since its identification in Australia in 2004, this species has caused substantial ecological and economic damage in several countries, including China, India, the United States, France, and Italy. Its capacity to hinder eucalyptus growth, particularly through severe damage to annual seedlings, has raised significant concerns about its impact on forestry and commercial eucalyptus production [17]. As *eucalyptus* serves as a key host, its distribution reflects the environmental conditions necessary for the species to establish. Consequently, changes in the cultivation range of eucalyptus are expected to directly affect the potential distribution and establishment of this invasive species.

While research has focused on its biological characteristics, occurrence patterns, and control measures, the global distribution and niche dynamics of *L. invasa* between its native and invasive ranges remain uncertain [18]. Despite research on the biological characteristics, distribution patterns, and control strategies of *L. invasa*, our understanding of the species ecological niche dynamics and geographical distribution on a global scale, particularly between its native and invasive ranges, remains limited [19]. Zhang et al. [17] applied the MaxEnt 3.4.1 model to predict the potential distribution of *L. invasa* in China under future climate change scenarios, revealing that suitable distribution areas may be concentrated in Yunnan, Guangxi, Guangdong, and Hainan, with a tendency to spread to higher latitudes such as Hubei, Anhui, Zhejiang, and Jiangsu. This highlights a significant expansion of the ecological niche for this species. Against the backdrop of climate fluctuations frequently induced by global climate change, investigating how various environmental factors affect the adaptability of *L. invasa* is crucial for improving the accuracy of pest outbreak predictions, developing effective control strategies, and comprehensively managing this harmful species. Such insights will provide a scientific basis for the construction of ecological niche models and the management of biological invasions.

MaxEnt, a widely used model for species distribution, was employed to predict the global invasion risk of *L. invasa* based on key environmental variables [20]. In this study, the optimized MaxEnt model was used to estimate the global distribution pattern of *L. invasa* and assess its niche dynamics. The objectives were threefold: (1) to compare the ecological niches of *L. invasa* in native and invasive habitats using the centroid shift, overlap, unfilling, and expansion (COUE) framework and PCA-env (Environmental PCA); methodologies; (2) to predict the potential areas of distribution of *L. invasa* at the global scale and their variation under future climate scenarios; and (3) to identify the key environmental variables that significantly influence these global distribution patterns.

## 2. Materials and Methods

### 2.1. Occurrence Records

Records of *L. invasa* occurrences in both invasive and native ranges were sourced from the following databases: Global Biodiversity Information Facility (GBIF. http://www.gbif.org/ (accessed on 6 May 2024)), Barcode of Life Data Systems (BOLD. http://www.boldsystem.org/ (accessed on 6 May 2024)), the Center for Agriculture and Bioscience (CABI. https://www.cabi.org/ (accessed on 6 May 2024)), Atlas of Living Australia (ALA. https://www.ala.org.au/ (accessed on 6 May 2024)), China National Knowledge Infrastructure (CNKI. http://www.cnki.net/ (accessed on 6 May 2024)), and Web of Science (https://www.webofscience.com/ (accessed on 6 May 2024)). Firstly, to mitigate sampling bias and improve data representativeness as well as the accuracy of model predictions, a 5-km resolution raster system was chosen as the analytical framework, guided by the spatial distribution characteristics of environmental variables. By refining the sampling point distribution, we ensured that each raster unit effectively represents its internal environmental conditions. Secondly, to optimize the dataset further and address biases stemming from over-concentrated or sparsely distributed samples, ENMTools v 1.3 was utilized for data processing. The software applies algorithmic optimization to limit each 5-square-kilometer raster unit to a maximum of one species occurrence record, thereby reducing the likelihood of certain regions being disproportionately emphasized in the model predictions. Finally, a data cleaning procedure was implemented to address recording biases and spatial autocorrelation. This involved identifying and eliminating duplicate records and excluding records linked to provincial capital cities. Such areas, heavily influenced by human activities, often exhibit a higher density of species records, potentially skewing the model’s predictions of natural ecological niches [21].

### 2.2. Environmental Data

Precipitation, temperature, and HII were selected as key environmental variables due to their significant influence on both habitat suitability and dispersal capacity of *L. invasa* [10]. Environment data were obtained from publicly accessible online sources. Near-present climate data (1970–2000) and altitude information, both at a resolution of 2.5 arcminutes, were sourced from WorldClim version 2.1 [22]. Future climate projections for the 2030s (2020–2040) and 2050s (2041–2060) at the same resolution were obtained from WorldClim v. 2.1, covering three Shared Socioeconomic Pathways (SSP1-2.6, SSP2-4.5, and SSP5-8.5) based on the BCC-CSM2-MR climate model. The datasets included 19 bioclimatic variables [22]. The HII is considered a valuable predictor because it reflects the extent of human-modified landscapes, which often facilitate the spread of invasive species through anthropogenic pathways such as transportation and land-use change. The HII data, with a 30-s resolution, were derived from the Global Human Influence Index (Geographic) v2 dataset for the years 1995–2004. ArcGIS (version 10.8) was used to standardize the spatial resolution of all environmental variables to 2.5 arcminutes. ENMTools was employed to detect and mitigate the issue of multivariate collinearity, which can impact SDM accuracy [21]. The contribution rates were derived using MaxEnt, while the correlation coefficients between pairs of variables were calculated using EnmTools. When the absolute value of the correlation coefficient between two variables exceeded 0.8, the variable with the higher contribution rate was retained. As a result, eight environmental variables were selected: mean diurnal range (bio2), maximum temperature of the warmest month (bio5), minimum temperature of the coldest month (bio6), annual precipitation (bio12), precipitation of the driest month (bio14), precipitation seasonality (bio15), altitude, and HII.

### 2.3. Niche Comparisons Between Native and Invasive Ranges

The PCA-env and COUE frameworks were employed to further analyze the ecological niches of *L. invasa* in both its native and invasive ranges, as these methods have proven effective in quantifying niche dynamics of invasive species [23,24]. The “ecospat” package in R (version 4.2.3) was used to assess the occurrence density, niche overlap, and niche similarity of *L. invasa* in both native and invaded regions [9]. In this study, species distribution data were projected onto the first two axes of the environmental space defined by Principal Component Analysis (PCA). This approach allowed us to compute the environmental space delineated by the first two PCA axes. A kernel smoothing function was then employed to estimate the actual distribution density and the available environmental space within the entire environmental background, thereby correcting potential sampling bias. Niche overlap was quantified using Schoener’s D metric, which ranges from 0 (no overlap) to 1 (complete overlap) [25]. This metric was calculated based on both distribution data and environmental variables. To account for environmental influences, PCA axes derived from environmental variables were incorporated into the analysis, providing insights into how these variables shape the ecological niche of *L. invasa*. Additionally, a niche similarity test was conducted to determine whether the observed similarity exceeded what would be expected by random distribution shifts [21]. After 1000 repetitions, the observed *D* value surpassed the 95% confidence interval of simulated values, indicating a statistically significant similarity between the niches in the native and invasive ranges [9,26]. Niche dynamics, including niche expansion, stability, and unfilling, were also quantified. This integrated methodology enabled a comprehensive assessment of niche overlap by combining both distribution data and environmental variables, offering a detailed understanding of the species’ response to environmental changes.

### 2.4. Potential Geographical Distribution of L. invasa

The MaxEnt model, a widely used and highly accurate niche model, was selected to predict suitable habitat areas for *L. invasa* in both its native and invasive ranges under current and future climate conditions [27,28]. Feature combinations (FC) and regularization multipliers (RM) are considered essential parameters for achieving optimal settings during model calibration [29]. RM values ranging from 0.5 to 4, in 0.5 increments, were tested. For the FC parameters, the Maxent model offered five types of features: linear (L), quadratic (Q), hinge (H), product (P), and threshold (T); and six combinations of these features were utilized: L, LQ, H, LQH, LQHP, and LQHPT (Phillips et al. 2006 [27]). The “ENMeval” R package was used to optimize these parameters, selecting the model with the lowest Delta Akaike Information Criterion corrected (AICc) to balance complexity and goodness of fit [29,30]. For the optimal MaxEnt model, 25% of occurrence records were used for testing, while 75% were used for training. Model performance was assessed using up to 10,000 background points, and accuracy was evaluated based on the true skill statistic (TSS) and the area under the receiver operating characteristic curve (AUC), with higher TSS and AUC values indicating better accuracy [31]. The final results were based on the maximum value obtained from 10 replicates. The model predictions were imported into ArcGIS, converted to raster files, and classified into four suitability classes (unsuitable, low, moderate, and high) using the maximum test sensitivity plus specificity logistic threshold. Spatial variations in potential geographic distribution were analyzed in ArcGIS based on three categories: unchanged, decreased, or increased.

## 3. Results

### 3.1. Global Occurrence of L. invasa

**Figure 1:**
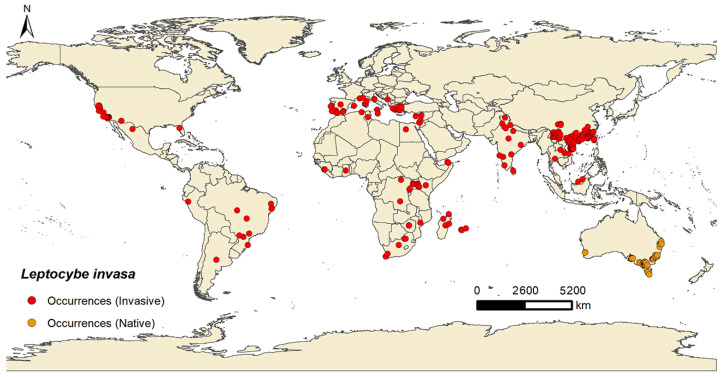
The occurrence records of *Leptocybe invasa* around the world.

The data concerning the global occurrence of *L. invasa* were divided into two distinct categories: the native range within Oceania and the invasive range, which spans North America, South America, Europe, Asia, and Africa (Figure 1). A total of 296 occurrence records were retained for modeling the potentially suitable habitats for *L. invasa* worldwide, including 70 records from the native range and 226 records from the invasive range.

### 3.2. Parameters and Performance of Optimal MaxEnt Models

**Figure 2:**
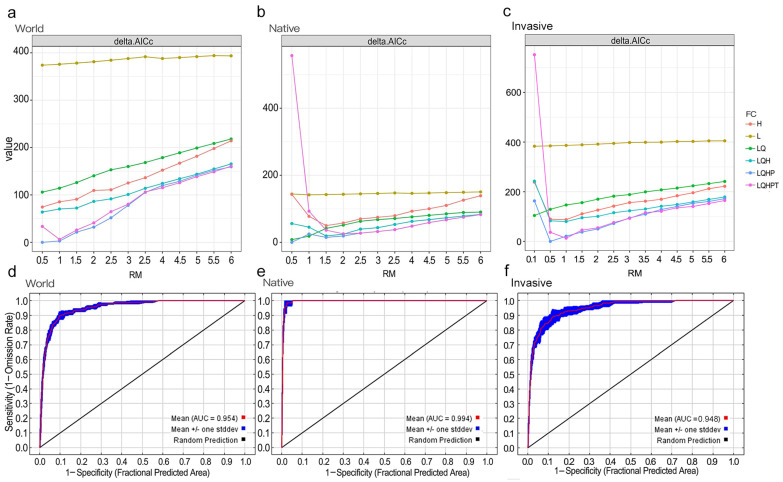
Delta AICc values of candidate models for *Leptocybe invasa*. (**a**) based on bioclimatic variables and occurrence records worldwide; (**b**) based on bioclimatic variables and occurrence records in the native range; (**c**) based on bioclimatic variables and occurrence records in the invasive range; (**d**) AUC values of the optimal model at the global scale; (**e**) AUC values of the optimal model in the native range; (**f**) AUC values of the optimal model in the invasive range.

The optimal MaxEnt models were selected based on the minimum Delta AICc values, as illustrated in the model calibration results (Figure 2 and Figure S1). For both the worldwide (invasive) and native ranges of *L. invasa*, the optimal models were based on bioclimatic variables and occurrence records, with FC set to LQHP and an RM value of 0.5. The optimal models demonstrated high predictive accuracy, with mean AUC values of 0.954 for the worldwide range, 0.994 for the native range and 0.948 for the invasive range. Similarly, the mean TSS values were 0.802 for the worldwide range, 0.937 for the native range and 0.807 for the invasive range, further underscoring the reliability of the model predictions.

### 3.3. Importance of Environmental Variables and Response Curve

**Figure 3:**
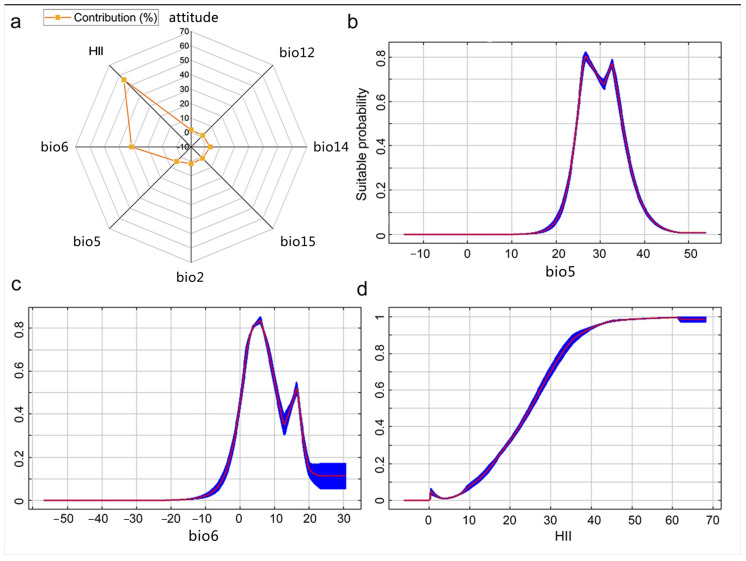
Contributions of environmental variables affecting the distribution of *Leptocybe invasa*. (**a**) and the response curves of important environmental variables (**b**–**d**). bio5: maximum temperature of the warmest month, bio6: minimum temperature of the coldest month, and HII: human influence index.

The contributions of environmental variables indicated that temperature and human influence variables were important environmental variables that influenced the distribution of *L. invasa*, including maximum temperature of the warmest month (bio5), minimum temperature of the coldest month (bio6), and human influence index (HII) (Figure 3a). The response curves of bio5 and bio6 indicated that the suitable probability of *L. invasa* presented a trend that first increased and then decreased. The response curves of bio5 and bio6 indicated that the suitable probability of *L. invasa* presented a trend that first increased and then decreased (Figure 3b,c). With an increase in the human influence index, the suitable probability of *L. invasa* presented an increased trend (Figure 3d).

### 3.4. Niche Comparison and Predicted Niche Occupancy Profiles

**Figure 4:**
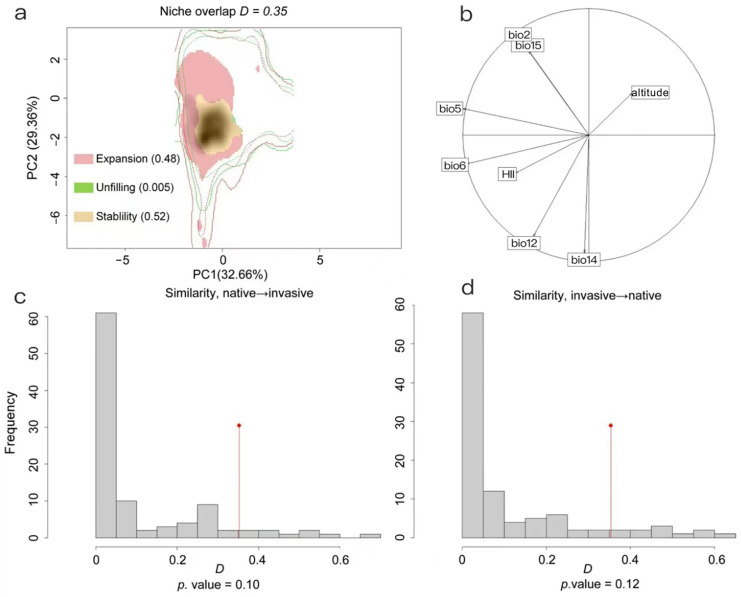
Niche overlap, similarity tests, and rates of contribution of bioclimatic variables between the native and invasive ranges of *Leptocybe invasa*. (**a**) Niche overlap; (**b**) contribution rates of environmental variables; (**c**,**d**) niche similarity tests. Histograms represent the null distribution of *D* obtained from 1000 iterations, which were compared to the observed Schoener’s *D* metric (red diamond) to assess niche similarity based on the tests comparing native to invasive (**c**) and invasive to native (**d**) ranges.

The first principal component (PC1) and the second principal component (PC2) explained 62.02% of the total variability, with the first principal component contributing 32.66% and the second principal component accounting for 29.36% (Figure 4a). Based on the COUE framework, the indexes for expansion, stability, and unfilling were found to have values of 0.48, 0.52, and 0.005, respectively (Figure 4a). Niche comparison revealed a low level of niche overlap between the native and invasive ranges (Schoener’s *D* = 0.35). Furthermore, the results of two similarity tests-one comparing the native to the invasive range and the other switching the two terms of comparison-showed a lack of similarity between these niches (*p* = 0.10 for native-to-invasive, *p* = 0.12 for invasive-to-native; Figure 4c,d). This indicated a substantial niche expansion in the invasive range, consistent with the results of the niche comparison test. The top three environmental variables contributing the most to the models were maximum temperature of the warmest month (bio5), minimum temperature of the coldest month (bio6), and HII (Figure 4b).

**Figure 5:**
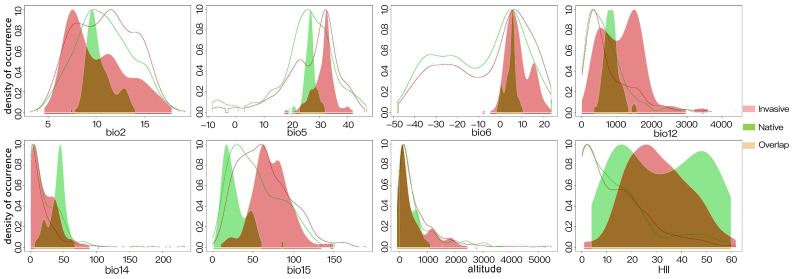
Predicted niche occupancy profiles based on the environmental variables incorporated in the models. bio2: Mean diurnal range, bio5: maximum temperature of the warmest month, bio6: minimum temperature of the coldest month, bio12: annual precipitation, bio14: Precipitation of the driest month, bio15: precipitation seasonality, altitude, and HII: human influence index. The green and red lines represent the density of occurrence of native and invasive ranges, respectively.

Niche occupancy profiles are used to describe and analyze the distribution and adaptability of species across specific environmental variables, such as climatic conditions. They predict the probability of species suitability across various environmental gradients through modeling, aiding in the comparison of ecological niche differentiation and overlap among species. The predicted niche occupancy profiles suggested that the populations of *L. invasa* in the invasive range were more adapted to a higher mean diurnal range and elevated temperatures than those in the native range (Figure 5). Furthermore, as indicated by annual precipitation (bio12), precipitation of the driest month (bio14), and precipitation seasonality (bio15), the invasive populations demonstrated a greater adaptability to the monsoonal climate patterns of the regions where they became established, which are characterized by substantial variations in precipitation throughout the year. Differences were detected between the invasive and native ranges in terms of the HII and elevation; however, in the latter case, they were not significant.

### 3.5. Prediction of Potentially Suitable Areas for L. invasa Under Near-Current and Future Climatic Scenarios

**Figure 6:**
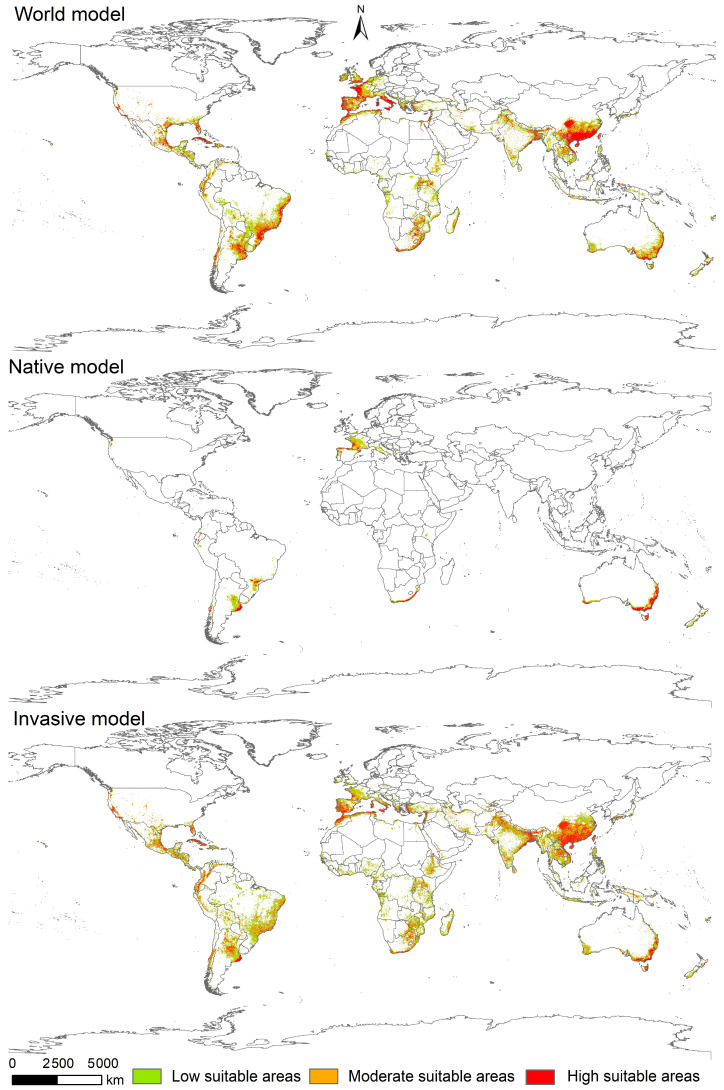
Potentially suitable areas for *Leptocybe invasa* at the global scale under near-current climate conditions based on global, native and invasive occurrence records.

The potentially suitable areas for *L. invasa* were predicted using MaxEnt models (Figure 6). According to the prediction based on the native range model, the current potential distribution of this species in the Northern Hemisphere covers the western coastal regions of Europe. In the Southern Hemisphere, it extends along the eastern coasts of Brazil and Argentina in South America, the Cape of Good Hope region in Africa, and the eastern coast of Australia in Oceania. Based on the prediction of the global model, the current potentially suitable habitats for *L. invasa* in the Northern Hemisphere were located along the coastal regions of the Gulf of Mexico in North America and Cuba and in the western regions and Mediterranean coastal areas of Europe, and those in the Southern Hemisphere were located in southern China and Southeast Asia, in the eastern and parts of the western coastal regions of South America, in regions south of the Equator in Africa, along the eastern and parts of the western coasts of Australia in Oceania as well as in New Zealand. Compared to the native range model, the global model predicted a significant expansion of the current potentially suitable habitats.

**Figure 7:**
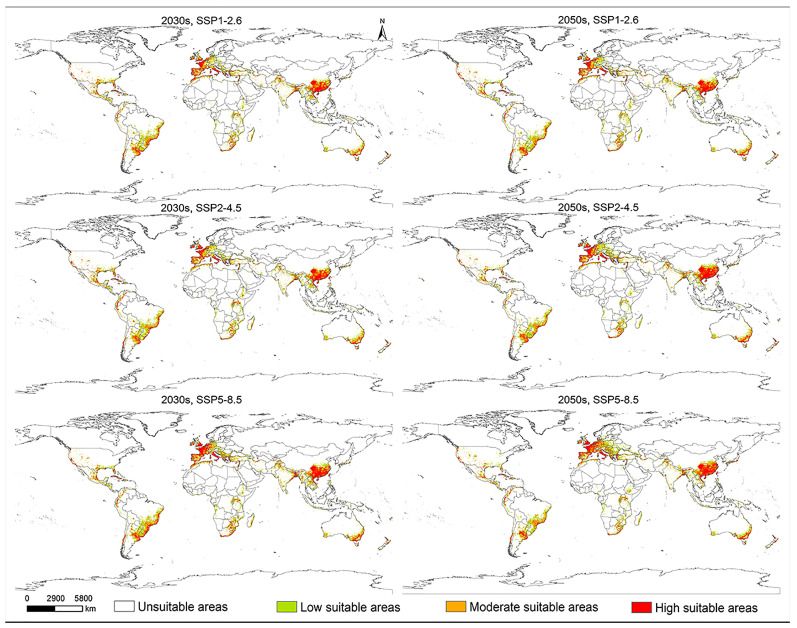
Potential geographical distribution of *Leptocybe invasa* under future climate scenarios (2030s and 2050s).

Under future climate scenarios, the highly suitable areas for *L. invasa* were shown to be primarily concentrated along the coasts of the Gulf of Mexico in North America, the western and Mediterranean coastal regions of Europe, southern China and Southeast Asia, the eastern coastal region of Brazil in South America, the southern coastal regions of Africa, and the eastern and western coastal regions of Australia in Oceania (Figure 7).

**Figure 8:**
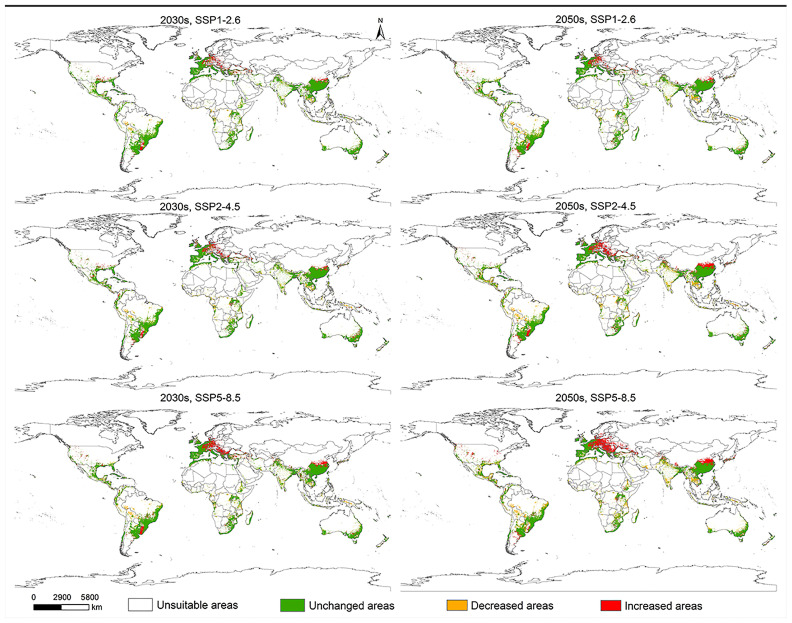
Changes in the potentially suitable areas for *Leptocybe invasa* at the global scale under future climate scenarios (2030s and 2050s).

Under future climate scenarios, the regions where *L. invasa* may expand due to the increase in potentially suitable habitats are primarily concentrated in central Europe, the Huaihe River region of China in Asia, and Uruguay in South America. In contrast, the regions with reduced suitable areas are primarily concentrated in Southeast Asia, central Africa, central South America, and the northeastern region of Australia in Oceania (Figure 8).

## 4. Discussion

### 4.1. Suitable Environmental Conditions

Globally, the habitat range of the invasive species *L. invasa* has significantly expanded primarily because of the combined effects of multifaceted factors. First, the increase in temperature resulting from global climate change has rendered previously cold or marginal regions more suitable for *L. invasa*, providing favorable conditions for its expansion into new habitats and for the extension of its survival range [17]. Temperature is a crucial factor influencing developmental timing and fecundity in insects [32]. Our findings indicated that mean diurnal range (bio2), maximum temperature of the warmest month (bio5), and minimum temperature of the coldest month (bio6) were significant parameters influencing the global distribution patterns of *L. invasa.* Second, variations in precipitation amounts and precipitation patterns, particularly the increased humidity in certain regions, have provided *L. invasa* with more suitable habitats and breeding conditions, facilitating the growth and expansion of its population. Previous studies have revealed that this species exhibits tolerance to low temperatures in the regions it invades [33,34].

This indicates that the niche of *L. invasa* in the invasive range may differ from that in the native range, highlighting its potential for invading regions located further north. Another study found that *L. invasa* adults in *eucalyptus* forests in southern Jiangxi, China, have various peaks of emergence from April to August (i.e., three primary and one secondary) with gall formation peaks lagging by 20–30 days, occurring mainly in May, July, and September. The insect undergoes 3–4 generations annually with significant overlap and a damage peak from June to September [35]. These studies suggest that warm climatic conditions favor the development of *L. invasa* populations, and temperatures during the warmest quarter may significantly impact its distribution and survival. The Mediterranean climate, which is characterized by dry and hot summers, allows the succession of more generations of this species each year compared to other climate types [36]. Our findings indicated that the suitable areas for *L. invasa* are located in the Mediterranean regions and coastal areas worldwide. When this species spreads to a region with suitable climatic conditions for survival and abundant food sources (e.g., a forest), it reproduces at an extremely high rate, particularly under high-temperature and high-humidity conditions. As a result, the population rapidly increases, facilitating widespread outbreaks and causing explosive damage [37]. Furthermore, *eucalyptus* plantations, due to their monoculture nature and low ecological diversity, are particularly prone to major outbreaks of *L. invasa*, and human activities have significantly facilitated the global spread of this species [18]. Our study has shown that as the HII increases, so does the probability of *L. invasa* occurrences. The boom in international trade and convenient transportation have enabled the easy movement of goods and hitchhiking insects attached to them across geographical boundaries, which has resulted in the inadvertent introduction of alien species into new regions [38]. Additionally, urbanization and changes in land use have not only reshaped natural landscapes but also created new ecosystems where invasive species can survive [39]. *Eucalyptus* species, the primary hosts of *L. invasa*, are widely cultivated globally due to their economic importance, and the human-mediated transportation of *eucalyptus* timber serves as the primary route for the dissemination of this invasive species [40]. Consequently, human factors play a major role in the distribution and survival of *L. invasa*. In summary, the expansion of its habitat range at the global scale is the result of the combined effect of multiple factors, including climate change, alterations in precipitation patterns, and human activities. This phenomenon not only poses a potential threat to local ecosystems but also presents new challenges for global biodiversity conservation. Consequently, it is of paramount importance to strengthen the monitoring, management, and research of invasive species as well as to develop effective prevention and control strategies.

### 4.2. Niche Expansion Dynamics and Prospective Changes in Distribution

By analyzing the climatic niche of *L. invasa* during the process of invasion, it was revealed that it has expanded to varying degrees in invaded sites worldwide (in Europe, North America, South America, Asia, and Africa) compared with its native habitat, which indicated that this species has already adapted to those new environments. When species migrate to new regions with environmental conditions similar to those in their native habitats, the likelihood of successful establishment significantly increases [41]. Under the influence of climate change, the geographical distribution and ecological niche of *Solenopsis invicta* Buren reveal that its ecological niche in its native region is not the same as in China [42]. By estimating the potentially suitable areas for *M. aquaticum* on a global scale, it was found that the ecological niche of *M. aquaticum* has expanded between its native and invasive ranges [10]. This study specifically evaluated the niche dynamics of *L. invasa* during the global invasion process. It was revealed that while the climatic conditions in the native environment of Australia are relatively uniform (which limits habitat diversity), the niche in the invaded regions exhibited notable variations, resulting in a low degree of niche overlap with the native range [43]. This phenomenon was validated using the MaxEnt model to predict the current and future suitable habitat areas for *L. invasa* in Oceania and various invaded regions globally (North America, South America, Europe, Africa, and Asia) under different climatic conditions. Furthermore, by comparing the current model-predicted distribution range with the distribution data provided by the European and Mediterranean Plant Protection Organization (EPPO), it was found that the model-predicted suitable habitats generally align with the actual distribution areas recorded by EPPO in overall trends, albeit with some regional discrepancies. These discrepancies may reflect the incomplete filling of ecological niches or limitations in data recording, providing valuable insights for further research into the invasion dynamics of *L. invasa* and its impact on regional ecosystems.

The model predictions indicated that, with the continuous changes in the global climate, particularly the increase in greenhouse gas emissions and the global temperature rise, the habitat range of *L. invasa* is expected to continue to expand, foreshadowing further economic and ecological losses. Currently, the areas facing a high risk of invasions by *L. invasa* are concentrated in East Asia, Southeast Asia, and Western Europe, where *eucalyptus* trees are widely planted or represent the basic resource for timber industries. By the 2030s and 2050s, central Europe and Brazil are projected to become new potential hotspots for *L. invasa* invasion. Given the reliance of these regions on the *eucalyptus* industry, it is of paramount importance to strengthen early warning and monitoring efforts. However, it is noteworthy that the suitable habitat range of *L. invasa* predicted by the model may be wider than the actual one. This is because, in addition to climatic factors, the presence of natural enemies, the adaptability to new environments, geographical barriers, and various other factors also profoundly influences the spatial distribution of invasive species [44,45]. Nonetheless, climate is a crucial factor affecting species distribution, and predicted variations in it still provide an essential theoretical basis for effectively preventing and controlling invasive species. In summary, this study combined species distribution models with niche dynamics analysis to estimate the potentially suitable areas for *L. invasa* at the global scale and compared niche differences between native and invaded regions. The results obtained not only provide scientific guidance for selecting low-risk areas for planting *eucalyptus* trees worldwide but also deepen our understanding of how climate change affects the global distribution patterns of *L. invasa*. Furthermore, these findings offer invaluable insights for future early warning, prevention, control, and management efforts against invasions by this species.

*Eucalyptus* trees, which are globally widespread, originate primarily from a limited number of specific ecological niches characterized by relatively stable and constrained climatic and soil conditions [46]. Nevertheless, with the expansion of human activities and the boom of international trade, *eucalyptus* species have been introduced into numerous new climatic regions, ranging from tropical rainforests to temperate grasslands and even frigid high-altitude zones, leaving their growth footprint across the globe [47]. This extensive introduction underscores the remarkable ecological adaptability and robust growth of *eucalyptus* trees. In the above-mentioned new environments, these trees exhibit astonishing growth rates and reproductive capacities, swiftly becoming a pivotal component of local ecosystems [17]. However, this rapid expansion also provides the ideal conditions for the establishment of alien pests such as *L. invasa*, which thrive in the newly accessed territories. Compared with the native habitats, the invaded regions, with their diverse and complex climatic characteristics, provide favorable conditions for the expansion of the ecological niche of pests like *L. invasa*. These insects adapt quickly to new environments, using *eucalyptus* trees as their primary host. They engage in parasitism by laying eggs in the trees, with the larvae feeding on the tree’s tissues, weakening its structure and vitality. Additionally, they consume sap and foliage, disrupt the tree’s nutrient flow, and introduce pathogens, all of which contribute to severe damage to the tree’s health and growth [48,49]. As their populations expand and distribution ranges extend, the threats posed to *eucalyptus* ecosystems intensify [50]. The expansion of the niche of *L. invasa* not only directly impacts *eucalyptus* tree growth and timber yield but also affects biodiversity [51]. Pest proliferation disrupts the competitive relationships between *eucalyptus* trees and other plants, affecting biodiversity [52]. Moreover, *L. invasa* may harbor pathogens or parasites, posing potential risks to wildlife that depends on *eucalyptus* resources and to human health [53].

To address the challenges posed by *L. invasa* invasions, comprehensive measures are imperative. Rigorous quarantine of imported nursery stock is essential to prevent its spread. Implementing forestry cultivation practices at various growth stages of *eucalyptus* forests is recommended. Since the larvae, pupae, and adults of *L. invasa* overwinter within galls, controlling their numbers can be achieved by collecting and destroying infested eucalypt branches. Employing biological control strategies, such as introducing natural predators, is advised. Planting *eucalyptus* species with high resistance can also reduce economic damage. This work provides essential elements for strategies of governments of different countries to urgently create and implement effective management plans to minimize risks with this pest, particularly in territories invaded by it.

## Data Availability

The data presented in the study are available in the paper.

## Supplementary Materials

The following supporting information can be downloaded at: https://www.mdpi.com/article/10.3390/insects15120985/s1, Figure S1:The values of the True Skill Statistic (TSS) for the world, native and invasive models of *Leptocybe invasa*.
