# Supplementary material for: Niche Expansion Has Increased the Risk of Leptocybe invasa Fisher Et LaSalle Invasions at the Global Scale
Source: Insects. 2024 Dec 12;15(12):985. doi: 10.3390/insects15120985 (PMC11676206; doi:10.3390/insects15120985)
Supplement: Supplementary file 1 [file insects-15-00985-s001.zip › insects-3326134-supplementary.pdf]

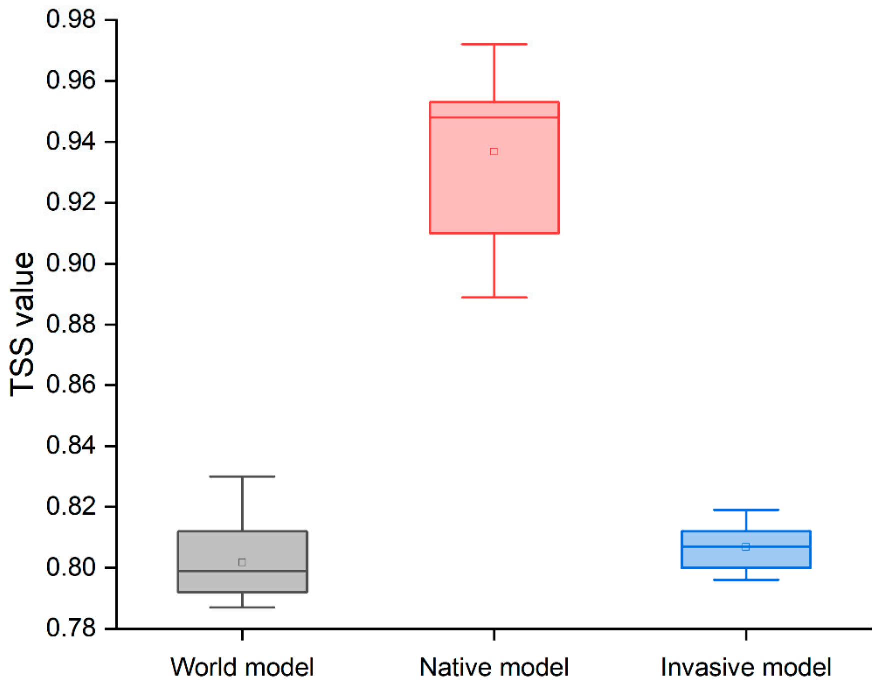

Figure S1. The values of the True Skill Statistic (TSS) for the world, native and invasive models of *Leptocybe invasa*.
